# Validation of a Modified Submaximal Balke Protocol to Assess Cardiorespiratory Fitness in Individuals at High Risk of or With Chronic Health Conditions—A Pilot Study

**DOI:** 10.3389/fspor.2021.642538

**Published:** 2021-04-22

**Authors:** Gert Sander Hamre Eike, Eivind Aadland, Ellen Eimhjellen Blom, Amund Riiser

**Affiliations:** Department of Sport, Food and Natural Sciences, Faculty of Teacher Education, Arts and Sports, Western Norway University of Applied Sciences, Sogndal, Norway

**Keywords:** submaximal test, estimation, maximal oxygen consumption, aerobic fitness, healthy life centers

## Abstract

**Objectives:** This study aims to validate a submaximal treadmill walking test for estimation of maximal oxygen consumption (VO_2max_) in individuals at high risk of or with chronic health conditions.

**Method:** Eighteen participants (age 62 ± 16 years; VO_2max_ 31.2 ± 5.9 ml kg^−1^ min^−1^) at high risk of getting or with established chronic diseases performed two valid modified Balke treadmill walking protocols, one submaximal protocol, and one maximal protocol. Test duration, heart rate (HR), and rate of perceived exertion (RPE) were measured during both tests. VO_2max_ was measured during the maximal test. VO_2max_ was estimated from the submaximal test by multiple regression using time to RPE ≥ 17, gender, age, and body mass as independent variables. Model fit was reported as explained variance (*R*^2^) and standard error of the estimate (SEE).

**Results:** The model fit for estimation of VO_2max_ from time to RPE ≥ 17 at the submaximal test, body mass, age, and gender was *R*^2^ = 0.78 (SEE = 3.1 ml kg^−1^ min^−1^, *p* ≤ 0.001). Including heart rate measurement did not improve the model fit.

**Conclusions:** The submaximal walking test is feasible and valid for assessing cardiorespiratory fitness in individuals with high risk of or chronic health conditions.

## Introduction

Cardiorespiratory fitness is a strong predictor of all-cause mortality and longer life expectancy (Kodama et al., 2009; Barry et al., 2014). The gold standard for assessing an individual's cardiorespiratory fitness is to directly measure their maximal oxygen consumption (VO_2max_) by the measurement of expiratory gases during maximal exercise (ATS/ACCP, 2003). VO_2max_ was first defined by Hill et al. (1924) as the oxygen consumption at a training intensity where oxygen intake reaches a plateau, despite an increase in workload. Thus, measurement of VO_2max_ is often performed during maximal graded exercise tests (Vehrs et al., 2007), where workload is increased until exhaustion. However, the direct measurement of VO_2max_ requires highly skilled personnel and sophisticated instrumentation and relies on participants' motivation and capability to push themselves to their physical limit.

When treadmill and cycle ergometer testing were first introduced into clinical practice, practitioners often used protocols such as the Balke protocol (Balke and Ware, 1959), from which VO_2max_ can be estimated from time to exhaustion (Pollock et al., 1982). A modified version of the Balke protocol has been used to assess cardiorespiratory fitness in a large sample of adults and elderly in Norway, where it was shown that VO_2max_ could be estimated reasonably well from time to exhaustion [*R*^2^ = 0.78, standard error of the estimate (SEE) = 4.6 ml kg^−1^ min^−1^] (Aadland et al., 2016). However, such maximal protocols may be less suitable for individuals with a variety of chronic health conditions (Lennon et al., 2012; Sartor et al., 2013), which has stimulated the development of submaximal tests for estimation of VO_2max_ (Jørgensen et al., 2009; Sartor et al., 2013).

In Norway, about half of the municipalities have established healthy life centers (HLCs) as a primary health care service to promote physical activity, smoking cessation, and a healthy diet among individuals at high risk of getting or with established chronic diseases (Ekornrud and Thonstad, 2018; Helsedirektoratet, 2019). The HLCs primarily recruit obese adults and older adults with multiple chronic conditions including musculoskeletal disorders and cardiovascular risk factors and diseases (Blom et al., 2019). The HLCs have applied a modified submaximal Balke walking treadmill protocol to asses cardiorespiratory fitness (Blom et al., 2020a), using Borg scale measured rate of perceived exertion (RPE) ≥ 17 (Borg, 1982) as the criterion for completing the test. The protocol was modified to suit the target group with multiple chronic conditions (i.e., with lower start speed than the original protocol) and for application with various types of treadmills with limited inclination and speed adjustment intervals. However, the validity of the submaximal protocol used in Norwegian HLCs has not been evaluated. Therefore, our aim was to validate this protocol in individuals at high risk of or with chronic health conditions against VO_2max_.

## Materials and Methods

### Design

In the present study, we performed two modified Balke protocols (Aadland et al., 2016; Blom et al., 2020a) on a motor-driven treadmill (Woodway PPS 55; WOODWAY GmbH, Weil am Rhein, Germany). The participants first performed a submaximal test that was terminated at RPE ≥ 17 and then a maximal test to exhaustion. The two tests were separated by a minimum of 4 days (12 ± 11) to allow the participants to recover between the tests.

### Patient and Public Involvement

During data collection in a previously published study (Blom et al., 2020b), employees at HLCs expressed the need for a valid tool to interpret and communicate HLC participants' cardiorespiratory fitness. Through communication with the HLC employees participating in the study (Blom et al., 2020a), the research question for the present study was developed. The local HLC recruited participants for the present study. The HLCs will be provided with the equation to estimate VO_2max_ for each participant undertaking the submaximal test and hence be able to interpret and communicate the participant's cardiorespiratory fitness by comparison with normal values.

### Participants

We recruited 23 adult participants (14 women and 9 men) aged 18–85 years taking part in exercise programs for individuals at high risk of or with established chronic disease(s) (i.e., HLC, union for heart and lung disease, cancer rehabilitation, and rheumatism habitation). The participants self-reported their risk factors for cardiometabolic diseases and known cardiometabolic diseases in a questionnaire previously used in other Norwegian studies testing cardiorespiratory fitness in HLCs (Blom et al., 2020a) and the general population (Aadland et al., 2016). Three participants (13%) had known heart disease, two (9%) occasionally felt chest pain at rest or when performing physical activity, five (22%) had hypertension, six (26%) used medication for hypertension or heart disease, and four (17%) had close relatives with heart infraction of sudden death before the age of 55 and 65 years for men and women, respectively. Three participants (13%) smoked regularly, six (26%) had hypercholesterolemia, and four had diabetes. Eight participants (35%) had none of the conditions mentioned above. Participants were not eligible if they had been advised by their physician to avoid heavy physical work. No potential participants volunteered for the study and were found not eligible. The test laboratory had access to a defibrillator, and an emergency room with a physician on call situated <1 km away provided medical backup. The study was approved by the Norwegian Centre for Research Data (reference number: 663351) and conducted according to the Helsinki declaration. All participants provided written informed consent and completed a health declaration prior to performing any tests. Information about the tests was given to each participant in a standardized manner.

### Tests

#### Submaximal Test Used in Norwegian HLC (Blom et al., 2020a)

Participants performed a self-paced treadmill familiarization prior to testing (speed 2.0–3.5 km h^−1^ for 2–7 min). The test started with a walking speed of 4.0 km h^−1^ on a flat treadmill. After 4 min, the treadmill inclination increased by 2% every minute until an inclination of 12%. We then increased the speed by 0.5 km h^−1^ every minute. The participants were asked to report RPE on the Borg 6–20 scale (Borg, 1982) 10 s before each increase in workload, and the test was terminated the first time an RPE ≥ 17 was reported. A progressive test protocol up to RPE 17 at the Borg scale has previously been shown to predict maximal oxygen uptake with reasonable accuracy compared to lower RPE (Coquart et al., 2014). Performance is reported as the time to RPE ≥ 17.

#### Maximal Test Previously Used in a Large Norwegian Study in Individuals ≥55 Years Old (Aadland et al., 2016)

A self-paced familiarization was performed prior to testing (speed 2.0–4.0 km h^−1^ for 2–7 min). The test started with a walking speed of 3.8 km h^−1^ at 2% inclination. After 4 min, inclination was increased by 2% each minute until it reached 20%. We then increased the speed by 0.5 km h^−1^ every minute until exhaustion. The test was terminated when participants were unable to keep up with the increasing workload despite verbal encouragement. Oxygen consumption was measured using the Moxus Modular VO_2_ system with mixing chamber and averaged over 30 s (AEI Technologies, Pittsburgh, Pennsylvania, USA), through a mask connected to a two-way valve (Hans Rudolph Inc., Kansas City, USA). The mask was thoroughly checked for any leaks prior to testing. VO_2_ was measured every 30th second and VO_2max_ was defined as the mean of the two highest, subsequent measured values. The VO_2max_ was considered valid if two of the three following criteria was achieved: (1) RPE ≥ 19, (2) respiratory exchange ratio (RER) ≥ 1.0, and (3) ≥97% of estimated heart rate maximal values (HR_max_). VO_2max_ is reported as milliliters per kilogram per minute. The end criteria was set to secure maximal intensity at test termination and to end up with a reasonable sample size. Besides VO_2max_, performance was measured using time to exhaustion and time to RPE ≥ 17 (for comparison with the submaximal test).

RPE and heart rate (HR) were measured continuously during both tests. HR was measured using a chest strap (Polar, Kempele, Finland). During the submaximal test, HR was displayed on the treadmill and measured every minute simultaneously with RPE, 10 s before increase in workload. During the maximal test, HR was measured through the Moxus software with a 30-s interval. RPE was assessed using the Borg 6–20 scale (Borg, 1982). HR is presented as the maximal values (HR_max_) and as a percent of estimated HR_max_ (HR_max_%) according to a previously published equation (211 – 0.64·age) (Nes et al., 2013). We measured height (without shoes) and body mass (with light clothing and no shoes) prior to the maximal test.

### Statistical Analysis

Data are presented as means and standard deviations (SD) unless stated otherwise. Associations between various measures of performance obtained from the two protocols are reported as the explained variance (*R*^2^). Differences between the included and excluded participants were tested with unpaired *t*-tests. We estimated VO_2max_ on the maximal protocol using multiple linear regression with time to RPE ≥ 17 at the submaximal protocol, age, body mass, gender, and HR_max_% as independent variables. The model fit is reported as the explained variance (*R*^2^) and standard error of the estimate (SEE). Assumption for linear regression was assessed with Kolmogorov–Smirnov and Shapiro–Wilk tests to test for normality in the dependent variable and collinearity between the independent variables; correlation between the independent variable was tested with Pearson's coefficient of correlation; normality of the residuals of the dependent variable was tested with plots (P-P and scatter); and residual statistics were assessed with standard residuals and Cook's distance. All analyses were performed using SPSS v. 25 (IBM Corporation, Software Group, Somers, NY).

## Results

**Figure 1:**
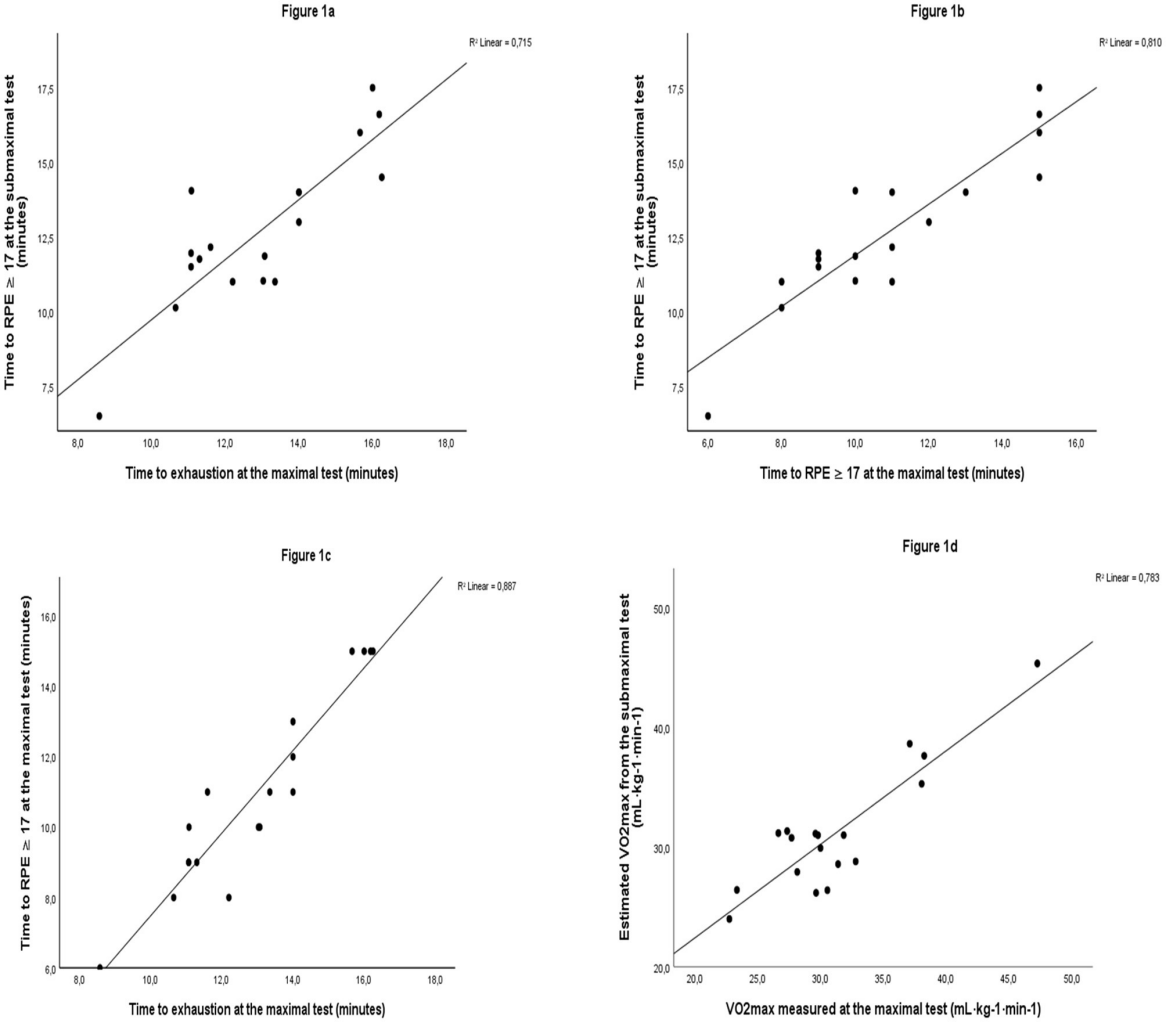
The correlation between time to RPE ≥17 at the submaximal test and time to exhaustion at the maximal test **(a)**, time to RPE ≥17 at the submaximal test and time to RPE ≥17 at the maximal test **(b)**, time to RPE ≥17 at the maximal test and time to exhaustion at the maximal test **(c)**, and estimated VO_2max_ and measured VO_2max_ **(d)** in individuals at high risk of getting or with established chronic diseases.

Of the 23 participants, one participant only performed the submaximal test and four were excluded for not meeting the present study's criteria for a valid VO_2max_ test (three had too low HR and RER and two had too low HR, RER, and RPE). Thus, the main analysis comprise18 participants aged 35–85 years (11 women and 7 men). Table 1 presents descriptive data and results from the exercise tests for both the included and the excluded participants. In addition to lower HR and RER, the excluded participants had lower performance at the maximal test and lower heart rate at the submaximal test. The association between time to RPE ≥ 17 at the submaximal test and time to exhaustion at the maximal test was *R*^2^ = 0.71, the association between time to RPE ≥ 17 at the submaximal test and time to RPE ≥ 17 at the maximal test was *R*^2^ = 0.81, and the association between time to exhaustion at the maximal test and time to RPE ≥ 17 at the maximal test was *R*^2^ = 0.89 (all *p* ≤ 0.001) (Figure 1).

**Table 1:** Descriptive data, results from cardiorespiratory fitness tests, and estimated VO_2max_ in individuals at high risk of getting or with established chronic diseases.

	**Women (*n* = 11)**	**Men (*n* = 7)**	**Total (*n* = 18)**	**Excluded 2♀and 2♂**
Age (years)	65 ± 12	57 ± 20	62 ± 16	72 ± 10
Weight (kg)	69.5 ± 13.7	92.6 ± 12.0	78.5 ± 17.2	86.4 ± 16.5
**Submaximal test (SMT)**				
Time to ≥17 RPE (min)	12.38 ± 2.73	13.20 ± 2.50	12.70 ± 2.60	10.06 ± 1.65
HR_max_ (bpm)	158 ± 16	157 ± 15	158 ± 16	132 ± 23.28^*^
HR_max_%	93 ± 10	90 ± 7	92 ± 9	81 ± 14
**Maximal test (MT)**				
Time to exhaustion (min)	12.60 ± 2.02	13.49 ± 2.49	12.95 ± 2.19	9.50 ± 1.85^*^
Time to RPE 17 (min)	10.36 ± 2.46	11.86 ± 3.08	10.94 ± 2.73	7.58 ± 1.65^*^
HR_max_ (bpm)	166 ± 17	169 ± 12	167 ± 15	142 ± 25^*^
HR_max_%	99 ± 5	99 ± 3	99 ± 4	87 ± 14.74^*^
RER	1.07 ± 0.09	1.05 ± 0.05	1.07 ± 0.08	0.93 ± 0.09^*^
VO_2max_ (ml kg^−1^ min^−1^)	29.84 ± 3.78	33.44 ± 8.09	31.24 ± 5.90	22.46 ± 3.07^*^
**Estimated VO**_**2max**_ **(ml kg**^**−1**^ **min**^**−1**^**)**				
Using SMT time, age, weight, and gender	29.80 ± 3.59	33.40 ± 6.81	31.21 ± 5.22	na
Using SMT time, age, weight, gender, and HR_max_%	29.85 ± 3.65	33.45 ± 6.84	31.25 ± 5.25	na

*HR_max_, maximal heart rate; bpm, beats per minute; HR_max_%, percent of estimated maximal heart rate; RPE, rate of perceived exertion; RER, respiratory exchange ratio; VO_2max_, maximal oxygen consumption; na, not applicable*;

* *p < 0.05 for the difference between the included and the excluded participants*.

The best model for estimation of VO_2max_ from the submaximal test was as follows: VO_2max_ = 45.873 + (1.159 submaximal test time) + (−0.301 body mass) + (−0.264 age) + (7.627 gender). This model showed strong association between estimated VO_2max_ and measured VO_2max_ (*R*^2^ = 0.78, SEE = 3.1 ml kg^−1^ min^−1^, *p* ≤ 0.001). Adding HR_max_% from the submaximal test to this model did not improve its performance (*R*^2^ = 0.79, SEE = 3.2, *p* ≤ 0.001).

As sensitivity analysis, we performed the analysis including both the 18 included participants and the four participants excluded for not meeting the present study's criteria for a valid VO_2max_ test, and the association between estimated VO_2max_ and measured VO_2max_ was identical (*R*^2^ = 0.79, SEE = 2.9, *p* ≤ 0.001).

VO_2max_ was normally distributed (*p* = 0.12/0.07), and there was no collinearity between the independent variables (*r* < 0.7); age (*r* = −0.5) and RPE ≥ 17 at the submaximal test (*r* = 0.6) were correlated with VO_2max_. Weight was not correlated to VO_2max_ (*r* = 0.1). Standard residuals ranged from −1.5 to 1.3 and Cook's distance ranged from 0.0 to 0.5.

## Discussion

This study shows that performance at the submaximal exercise test used to assess cardiorespiratory fitness among individuals at high risk of or with chronic health conditions at HLCs in Norway associated strongly with performance at the maximal test performed according to the gold standard for cardiorespiratory fitness testing. Thus, time to reach RPE 17 is a valid measure of cardiorespiratory fitness in this at-risk population and can be used to estimate VO_2max_ by the equation suggested herein.

The present study showed a strong association between estimated VO_2max_ from performance at the submaximal test and directly measured VO_2max_ in individuals at high risk of getting or with established chronic diseases. Pollock et al. (1982) and Vehrs et al. (2007) reported an even stronger association with lower SEE when estimating VO_2max_ from a graded maximal treadmill walk test in healthy women and for a submaximal treadmill jogging test at a self-selected pace in fit adults, respectively. However, the association found in the present study was similar to the ability of the maximal modified Balke treadmill protocol used to estimate VO_2max_ in a large Norwegian adult population (Aadland et al., 2016) and more accurate than a previously published model estimating VO_2max_ from a submaximal treadmill walking test in overweight children (Nemeth et al., 2009). Thus, we regard the submaximal walking protocol evaluated in the present study well-suited for the assessment of cardiorespiratory fitness in an adult clinical and subclinical population.

The submaximal test used at Norwegian HLCs does not require measurements of HR. However, we investigated if including HR_max_% during the submaximal test improved the model fit when estimating VO_2max_, based on the linear relationship between HR and oxygen consumption (Sartor et al., 2013). Yet, adding HR_max_% to the model did not improve the model. Given that the performance of both models was similar, adding measurements of HR for estimation of cardiorespiratory fitness is not necessary.

### Potential Bias

The mean number of days between the tests was 12 ± 11 days. This illustrates that some participants had several weeks from the submaximal test to the maximal. We have no information about the lifestyle or change in lifestyle within this period, and fitness may have changed between the two tests. For instance, if participants lost weight, stopped smoking, or became more physically active in the period between the tests, this change may have had an impact on their performance. Indeed, the time to RPE ≥ 17 at the maximal test was more strongly associated with measured VO_2max_ than time to RPE ≥ 17 at the submaximal test, which might indicate some day-to-day variation in fitness and/or change in fitness between the tests.

RPE is a simple, valid, and reliable means to quantify the subjective feeling of exercise tolerance and exertion (Kang et al., 1998; Doherty et al., 2001). Borg's scale relies on verbal anchors connected to the different scores of RPE, and it is possible that individuals at high risk of disease interpret the meanings of the verbal anchors differently than those used to construct the scale (Dawes et al., 2005). The participants in this study had little or no previous experience with exercise, use of Borg scale, and treadmills and lacked experience with strenuous exercise, which may have introduced a bias to the measurements. However, the population in the present study is comparable to participants in HLCs performing the tests as a part of their lifestyle change program. At the maximal test, the participants had already performed a walking test on a treadmill, and the difference in experience with strenuous treadmill walking may have introduced a systematic bias between the two tests. We could have randomized the order of the two tests or performed a familiarization test to reduce this systematic bias. However, we wanted to perform the submaximal tests as close as possible to the HLCs' test setting, as we believe that the bias introduced by familiarization to strenuous treadmill walking ahead of the submaximal test would be larger than the bias introduced by the fixed order of tests.

The duration of the maximal test was 13 ± 2.2 min; thus, it exceeded the traditionally accepted optimal VO_2max_ test duration of 10 ± 2 min (Ross, 2003). However, in their review, Midgley et al. (2008) found that treadmill protocols lasting between 5 and 26 min produced valid VO_2max_ measurements. Given that time to exhaustion from this study slightly exceeded the traditional 8–12-min duration and that Midgley et al. (2008) found that much shorter and longer treadmill protocols also produced valid VO_2max_ measurments indicate that the duration of our maximal test did not compromise the validity of the VO_2max_ measurement.

Participants were allowed to slightly support themselves if feeling pain or to maintain balance. However, supporting oneself during a submaximal test may increase time to “exhaustion” and thus lead to an overestimation of VO_2max_ (Manfre et al., 1994). In our study, participants supporting themselves during the submaximal test also supported themselves during the maximal test to standardize the test condition. Supporting oneself during direct measurement of VO_2max_ will probably not increase VO_2max_, as support during treadmill walking/running decreases the exercise load, and one must increase exercise load to increase VO_2_. Thus, support may create a bias in estimation of VO_2max_, as it might be overestimated in subjects supporting themselves and underestimated in subjects not supporting themselves. However, in a real-life test setting, some subjects with chronic health conditions will need to support themselves, and we chose to include both participants supporting themselves and those not supporting themselves to increase the external validity of our results.

Unfortunately, the questionnaire assessing risk factors for cardiometabolic diseases and known cardiometabolic diseases was used as eligibility screening, and the answers were not linked to the result from the tests; thus, we cannot link the results from the questionnaire to individuals. Six participants used medication for hypertension or heart disease. We do not know the type of medication. If someone took beta-blockers, it would probably blunt the heart rate response to the exercise tests (Wonisch et al., 2003). However, all included participants reached ≥97% of estimated HR_max_, and the performance of the model remained the same also when we included the participants not fulfilling our criteria for a valid VO_2max_ test. Thus, the use of beta blockers probably did not affect the results in the present study.

Submaximal tests for estimating VO_2max_ have shown to produce valid and reliable measures in different populations (Oja et al., 1991; Vehrs et al., 2007; Nemeth et al., 2009). Estimation using the UKK walk test (Oja et al., 1991) is a simple self-paced field test and can be applied in large groups of healthy adults, but it requires a large area of flat ground. Our findings suggest the submaximal protocol evaluated herein is a valid and feasible test. Since treadmills are accessible in most fitness facilities and laboratories, the test setting is easily standardized and not limited by climate or weather. Using the best-fit model from this study will allow HLCs to compare their participants with national reference values and ultimately define the health risk of this specific population.

### Strengths and Limitations

A strength of this study is the inclusion of a clinical or subclinical population with various health conditions, leaving direct evidence of test performance in this group for which the test is intended for use. Another strength is the strict criteria for verification of a maximal performance, ensuring that all included VO_2max_ measurements were valid. The appropriate criteria for VO_2max_ is highly debated (Edvardsen et al., 2014), and we opted for stricter criteria for VO_2max_ than the criteria used for testing the general population in Norway (Aadland et al., 2016). Our criteria for valid VO_2max_ may have been stricter than necessary as when we included the four subjects not meeting our criteria, the performance of the model remained unchanged. Our sample includes 18 subjects, which is lower than the minimum sample size for regression of 25 recommended by Jenkins and Quintana-Ascencio (Jenkins and Quintana-Ascencio, 2020). Thus, the low sample size reduces the power and generalizability of our findings. The data met all the tested assumptions for linear regression except for a correlation between VO_2max_ and age; however, age was a significant contributor in the model. Due to the small sample size, we could not perform a cross validation of our equation for estimation of VO_2max_. Thus, despite a relatively large variation in age, cardiorespiratory fitness, and health conditions among our participants, which strengthens the generalizability of our findings, the findings should be interpreted with caution, and future studies should seek to include a larger sample and perform a cross validation in a similar population.

## Conclusion

The present study found a strong association between performance on a submaximal treadmill walking protocol and measured VO_2max_ in a clinical or subclinical population. The study demonstrates that the submaximal walking test is valid and feasible as a means for assessing cardiorespiratory fitness in a population and in a setting where direct measurement of VO_2max_ is challenging. However, our findings need verification in a larger study sample.

## Data Availability Statement

The datasets presented in this article are not readily available because the subjects signed an informed consent stating that the data should be handled by the researchers conducting the study and deleted after the study was published according to the approval given by Norwegian Centre for Research Data. Requests to access the datasets should be directed to Amund Riiser, amund.riiser@hvl.no.

## Ethics Statement

The study was reviewed and approved by Norwegian Centre for Research Data (Reference No: 663351). The participants provided their written informed consent to participate in this study.

## Author Contributions

GE designed the study, collected the data, drafted the manuscript, and approved the final version. EA designed the study, planned the analysis, contributed to the manuscript, and approved the final version. EB designed the study, contributed to the manuscript, and approved the final version. AR planned the study, performed the analysis, and finalized the manuscript.

## Conflict of Interest

The authors declare that the research was conducted in the absence of any commercial or financial relationships that could be construed as a potential conflict of interest.
