# Correction: Validation of a modified submaximal Balke protocol to assess cardiorespiratory fitness in individuals at high risk of or with chronic health conditions—a pilot study

**DOI:** 10.3389/fspor.2026.1854460

**Published:** 2026-05-26

**Authors:** Gert Sander Hamre Eike, Eivind Aadland, Ellen Eimhjellen Blom, Amund Riiser

**Affiliations:** Department of Sport, Food and Natural Sciences, Faculty of Teacher Education, Arts and Sports, Western Norway University of Applied Sciences, Sogndal, Norway

**Keywords:** submaximal test, estimation, maximal oxygen consumption, aerobic fitness, healthy life centers

There was an omission of how gender was coded.

A correction has been made to the section **Materials and Methods**, *Statistical Analysis*. The corrected text appears below:

“We estimated VO_2max_ on the maximal protocol using multiple linear regression with time to RPE ≥ 17 at the submaximal protocol, age, body mass, gender, and HR_max_% as independent variables. In the regression model females were coded as 1, and males as 2.”

A correction has been made to the section **Results**, Paragraph 2. The corrected text appears below:

“The best model for estimation of VO_2max_ from the submaximal test was as follows: VO_2max_ = 45.873 + (1.159 submaximal test time) + (−0.301 body mass) + (−0.264 age) + (7.627 gender). (Females coded as 1, males as 2.)”

The original version of this article has been updated.

